# Supplementary material for: Impact of age and comorbidities on SARS-CoV-2 vaccine-induced T cell immunity
Source: Commun Med (Lond). 2023 Apr 24;3:58. doi: 10.1038/s43856-023-00277-x (PMC10124939; doi:10.1038/s43856-023-00277-x)
Supplement: Supplementary file 1 — Supplemental Materials [file 43856_2023_277_MOESM1_ESM.pdf]

SUPPLEMENTARY

Supplementary Table 1: Vaccine-related response for CD4+ and CD8+ T cells at day 90.

	Day 90 Spike Specific CD4+ T Cells				Day 90 Spike Specific CD8+ T Cells			
	Tertile 1	Tertile 2	Tertile 3	p-value	Tertile 1	Tertile 2	Tertile 3	p-value
Number of participants, n (%)	154 (33.3)	154 (33.3)	154 (33.3)		151 (33.3)	148 (33.3)	150 (33.3)	
**Baseline characteristics**								
Age at enrolment, median [Q1,Q3]	70 [57,75]	64 [52,69]	63 [51,69]	<0.001	68 [54,75]	62 [48,70]	65 [55,69]	0.02
Age group, n (%)								
<65	53 (24.8)	77 (36.0)	84 (39.3)	<0.001	62 (29.1)	78 (36.6)	73 (34.3)	0.009
65-74	43 (32.1)	49 (36.6)	42 (31.3)		38 (29.2)	44 (33.8)	48 (36.9)	
≥75	58 (50.9)	28 (24.6)	28 (24.6)		51 (48.1)	26 (24.5)	29 (27.4)	
Sex, n (%)								
Male	77 (34.4)	66 (29.5)	81 (36.2)	0.208	73 (34.0)	64 (29.8)	78 (36.3)	0.315
Female	77 (32.4)	88 (37.0)	73 (30.7)		78 (33.3)	84 (35.9)	72 (30.8)	
Vaccine type, n (%)								
BNT162b2	125 (52.1)	68 (28.3)	47 (19.6)	<0.001	115 (50.4)	66 (28.9)	47 (20.6)	<0.001
mRNA-1273	25 (11.7)	83 (39.0)	105 (49.3)		33 (15.6)	79 (37.3)	100 (47.2)	
ChAdOx1+mRNA	4 (44.4)	3 (33.3)	2 (22.2)		3 (33.3)	3 (33.3)	3 (33.3)	
Days between first and second dose, median [Q1,Q3]	23 [21,28]	35 [21,35]	35 [24,35]	<0.001	24 [21,35]	34 [22,35]	35 [23,35]	<0.001
Days from first vaccine to third study visit, median [Q1,Q3]	91 [89,93]	91 [88,93]	91 [87,96]	0.562	91 [89,94]	91 [89,95]	91 [88,93]	0.338
CCI, n (%)								
0	105 (29.9)	121 (34.5)	125 (35.6)	0.012	103 (30.1)	115 (33.6)	124 (36.3)	0.062
1-2	37 (40.7)	31 (34.1)	23 (25.3)		39 (44.8)	27 (31.0)	21 (24.1)	
>2	12 (60.0)	2 (10.0)	6 (30.0)		9 (45.0)	6 (30.0)	5 (25.0)	
Comorbidities in the previous 5 years, n								
Myocardial infarction	<5*	<5*	<5*	1.000	<5*	<5*	<5*	0.875
Congestive heart failure	5	5	<5*	0.826	7	<5*	<5*	0.367
Peripheral vascular disease	<5*	<5*	<5*	1.000	<5*	<5*	<5*	1.000
Cerebrovascular disease	8	5	6	0.769	<5*	6	8	0.474
Dementia								
Chronic pulmonary disease	10	<5*	<5*	0.168	8	<5*	6	0.548
Rheumatological disease	7	<5*	<5*	0.089	<5*	5	<5*	0.184
Peptic ulcer disease			<5*	0.332		<5*		0.108
Mild liver disease	5	<5*		0.106	6	<5*	<5*	0.089
Diabetes without chronic complications	8	<5*	5	0.551	6	5	6	1.000
Diabetes with chronic complications	<5*			1.000	<5*			1.000
Hemiplegia or paraplegia								
Any malignancy, including leukemia and lymphoma	20	14	21	0.442	22	18	12	0.182
Moderate or severe liver disease	<5*			0.036	<5*	<5*		0.280
Metastatic solid tumor	<5*		<5*	1.000	<5*		<5*	1.000
AIDS/HIV	<5*	<5*	<5*	0.913	<5*	<5*	<5*	0.651
Renal disease	<5*	<5*		0.379	<5*	<5*	<5*	0.626
Organ transplantation	8	10	8	0.905	14	6	6	0.107
**Day 90 immune response**								
Serological vaccine responder group, n (%)								
Hypo	18 (50.0)	11 (30.6)	7 (19.4)	<0.001	22 (61.1)	6 (16.7)	8 (22.2)	0.001
Moderate	55 (52.4)	29 (27.6)	21 (20.0)		40 (39.6)	38 (37.6)	23 (22.8)	
High	80 (25.2)	112 (35.2)	126 (39.6)		88 (28.5)	103 (33.3)	118 (38.2)	
Total SARS CoV-2 Spike IgG Antibodies ($\cdot 10^5$ AU/mL), median [Q1,Q3]	1.15 [0.48,3.20]	3.03 [1.35,4.65]	4.04 [1.76,4.68]	<0.001	1.30 [0.55,3.56]	3.16 [1.64,4.55]	3.95 [1.48,4.66]	<0.001
**Post day 90**								
Breakthrough Infection, n (%)								
Yes	48 (35.3)	37 (27.2)	51 (37.5)	0.183	43 (32.1)	49 (36.6)	42 (31.3)	0.568
No	106 (32.5)	117 (35.9)	103 (31.6)		108 (34.3)	99 (31.4)	108 (34.3)	
Follow-up days, median [Q1,Q3]	252 [236,266]	224 [196,257]	215 [194,251]	<0.001	249 [226,270]	223 [188,258]	216 [201,250]	<0.001

* Groups with small numbers (<5 participants per cell) where there is the potential that individual participants could be identified or be able to identify themselves have been edited to maintain participant confidentiality. Charlson Comorbidity Index (CCI).

Supplementary Figure 1: Timeline of vaccine doses and study visits.

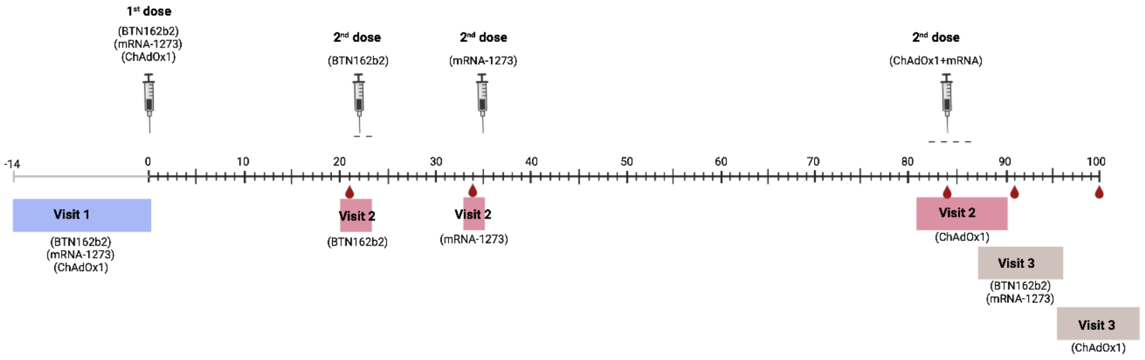

Vaccine doses are depicted above the timeline by syringes at the median value and horizontal dotted lines showing IQR. Study visits are depicted below the timeline by blood drops at median and boxes showing IQR. Day 0 was defined as the day of the first vaccine dose. The median number of days from first vaccine dose to visit 2 (day 21) was 21, 34, and 84 days for BNT162b2, mRNA-1273, and ChAdOx1, respectively. The median number of days from first vaccine dose to visit 3 (day 90) was 91 days for BNT162b2 and mRNA-1273, and 100 days for ChAdOx1. The figure was created with BioRender.com.

Supplementary Figure 2: Gating strategy in AIM assay.

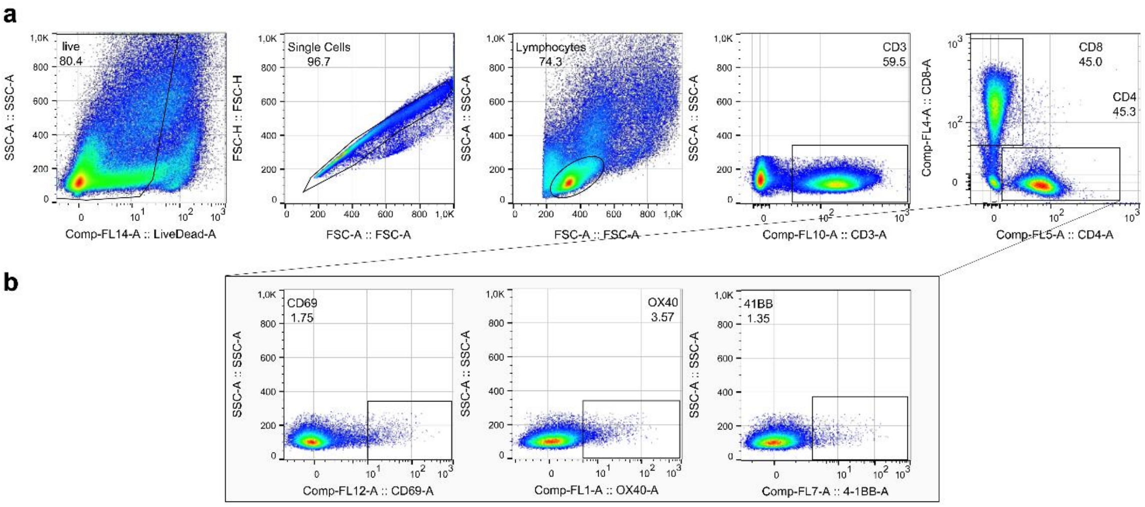

*(**a**) Gating strategy to identify CD4+ and CD8+ T cells. (**b**) Example of gating strategy for the thee AIMs (CD69, OX40, and 41BB) for CD4+ T cells; identical gating was done for CD8+ T cells. Figure created in FlowJo Layout Editor with representative substudy participant sample.*

Supplementary Figure 3: SARS-CoV-2 Spike-specific T cells with vaccine type and study visit.

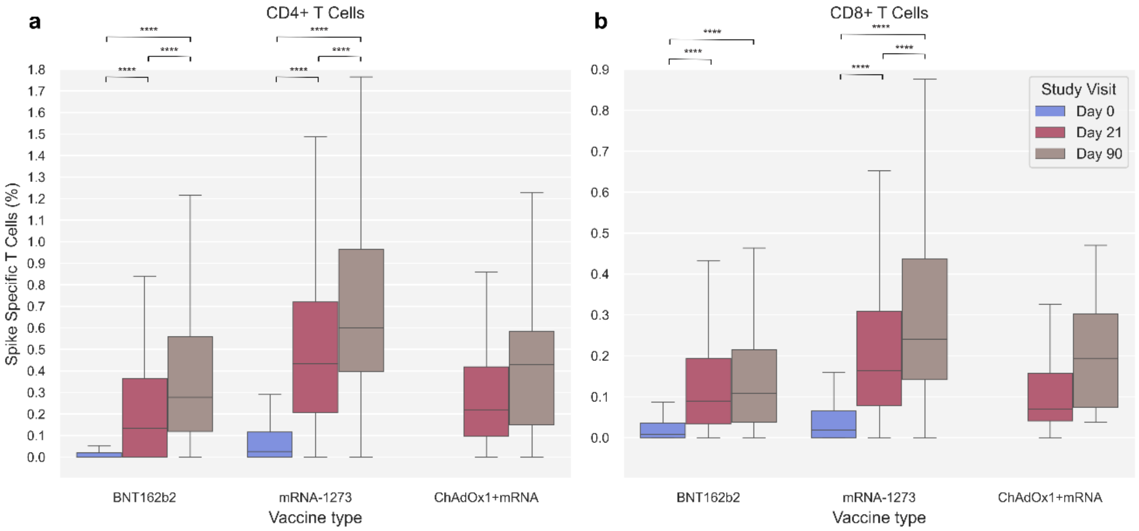

*SARS-CoV-2 Spike-specific CD4+ (**a**) and CD8+ (**b**) T cells at days 0 (blue), 21 (red), and 90 (brown) stratified by vaccine type. For CD4+ T cells, group sizes are; BNT162b2, n=127 (day 0), n=212 (day 21), n=240 (day 90); mRNA-1273, n=159 (day 0), n=197 (day 21), n=213 (day 90); ChAdOx1+mRNA, n=0 (day 0), n=51 (day 21), n=9 (day 90). For CD8+ T cells, group sizes are; BNT162b2, n=115 (day 0), n=198 (day 21), n=228 (day 90); mRNA-1273, n=157 (day 0), n=195 (day 21), n=212 (day 90); ChAdOx1+mRNA, n=0 (day 0), n=51 (day 21), n=9 (day 90). Data was compared using unpaired, non-parametric Mann–Whitney U test. Error bars show the distribution within 1.5 times IQR. p-value annotation legend: *: $1.00{\cdot}10^{-02} < p \leq 5.00{\cdot}10^{-02}$, **: $1.00{\cdot}10^{-03} < p \leq 1.00{\cdot}10^{-02}$, ***: $1.00{\cdot}10^{-04} < p \leq 1.00{\cdot}10^{-03}$, ****: $p \leq 1.00{\cdot}10^{-04}$.*

Supplementary Figure Supplementary Figure 4: Correlation between SARS-CoV-2 Spike CD4+ and CD8+ response.

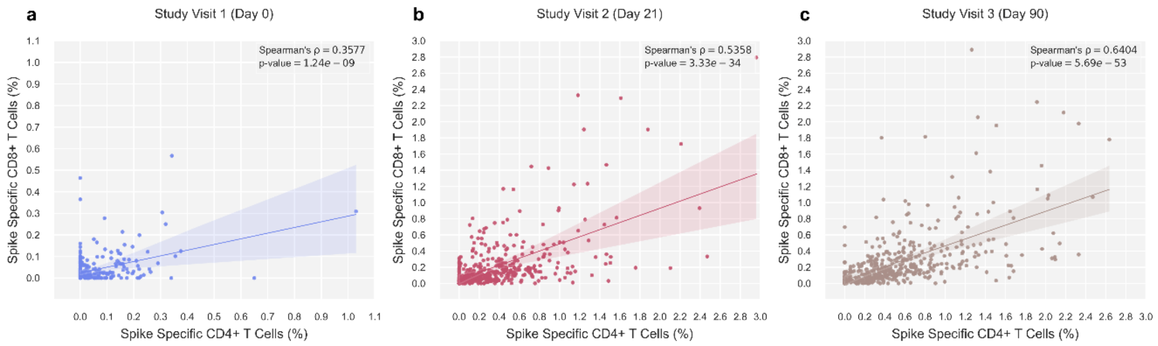

*Correlation between SARS-CoV-2 Spike-specific CD4+ (horizontal axis) and CD8+ (vertical axis) T cells at (**a**) day 0 (blue, n=272), (**b**) day 21 (red, n=442), and (**c**) day 90 (brown, n=447). Data was plotted with a linear regression model fit. Translucent bands around the regression line show the 99% confidence interval for the regression estimate. The confidence interval was estimated using a bootstrap.*

Supplementary Figure 5: SARS-CoV-2 Spike-specific T cells with age group and Charlson Comorbidity Index (CCI).

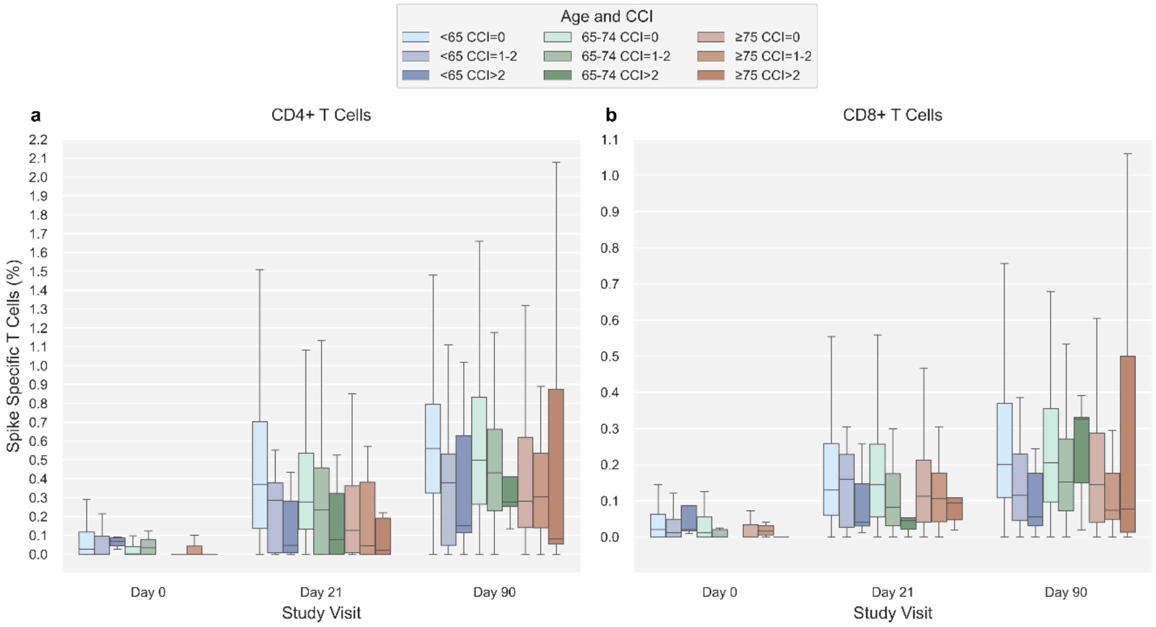

*SARS-CoV-2 Spike-specific CD4+ (**a**) and CD8+ (**b**) T cells at day 0, 21, and 90 stratified by age group (<65 years [blue], 65-74 years [green], ≥75 years [red]) and Charlson Comorbidity Index (CCI) (CCI=0 [light color], CCI=1-2 [medium color], CCI>2 [dark color]). For CD4+ T cells (left), group sizes are; at day 0 n=121 (<65 CCI=0), n=17 (<65 CCI=1-2), n=4 (<65 CCI>2), n=47 (65-75 CCI=0), n=7 (65-74 CCI=1-2), n=0 (65-74 CCI>2), n=81 (≥75 CCI=0), n=8 (≥75 CCI=1-2), n=1 (≥75 CCI>2); at day 21 n=195 (<65 CCI=0), n=28 (<65 CCI=1-2), n=11 (<65 CCI>2), n=74 (65-75 CCI=0), n=42 (65-74 CCI=1-2), n=7 (65-74 CCI>2), n=75 (≥75 CCI=0), n=23 (≥75 CCI=1-2), n=5 (≥75 CCI>2); at day 90 n=175 (<65 CCI=0), n=30 (<65 CCI=1-2), n=9 (<65 CCI>2), n=89 (65-75 CCI=0), n=40 (65-74 CCI=1-2), n=5 (65-74 CCI>2), n=87 (≥75 CCI=0), n=21 (≥75 CCI=1-2), n=6 (≥75 CCI>2).* For CD8+ T cells (left), group sizes are; at day 0 n=120 (<65 CCI=0), n=15 (<65 CCI=1-2), n=4 (<65 CCI>2), n=47 (65-75 CCI=0), n=7 (65-74 CCI=1-2), n=0 (65-74 CCI>2), n=71 (≥75 CCI=0), n=7 (≥75 CCI=1-2), n=1 (≥75 CCI>2); at day 21 n=195 (<65 CCI=0), n=27 (<65 CCI=1-2), n=11 (<65 CCI>2), n=72 (65-75 CCI=0), n=39 (65-74 CCI=1-2), n=7 (65-74 CCI>2), n=67 (≥75 CCI=0), n=21 (≥75 CCI=1-2), n=5 (≥75 CCI>2); at day 90 n=175 (<65 CCI=0), n=29 (<65 CCI=1-2), n=9 (<65 CCI>2), n=88 (65-75 CCI=0), n=37 (65-74 CCI=1-2), n=5 (65-74 CCI>2), n=79 (≥75 CCI=0), n=21 (≥75 CCI=1-2), n=6 (≥75 CCI>2). *Error bars show the distribution within 1.5 times IQR.*

Supplementary Figure 6: SARS-CoV-2 Spike-specific T cells at cay 90 stratified by breakthrough infections with B.1.617.2 (Delta) and B1.1.529 (Omicron) variants.

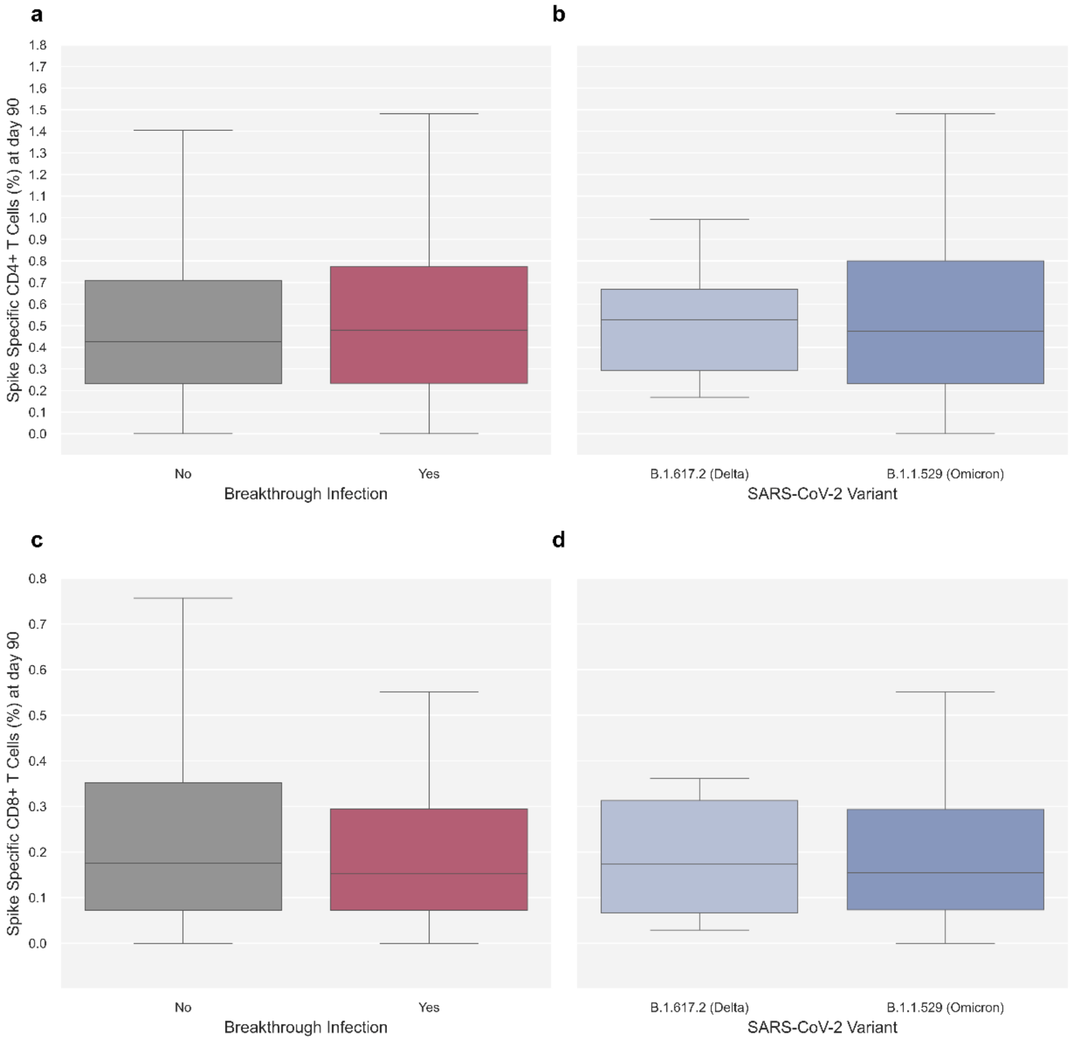

*A total of 167 breakthrough infections were recorded in the entire cohort of 655 participants; 1 unknown variant, 10 B.1.617.2 (Delta), and 156 B.1.1.529 (Omicron) variant. The figure shows breakthrough infections relative to SARS-CoV-2 Spike-specific CD4+ T cells (**a**, **b**), CD8+ T cells (**c**, **d**) at day 90. 136 of 462 participants with CD4+ T cell data experienced breakthroughs **(a)**; 10 Delta and 125 Omicron variant **(b)**. 134 of 449 participants with CD8+ T cell data experienced breakthroughs **(c)**; 10 Delta and 123 Omicron variant **(d)**. Data was compared using unpaired, non-parametric Mann–Whitney U test. Error bars show the distribution within 1.5 times IQR. p-value annotation legend: *: $1.00 \cdot 10^{-02} < p \leq 5.00 \cdot 10^{-02}$, **: $1.00 \cdot 10^{-03} < p \leq 1.00 \cdot 10^{-02}$, ***: $1.00 \cdot 10^{-04} < p \leq 1.00 \cdot 10^{-03}$, ****: $p \leq 1.00 \cdot 10^{-04}$.*
