# Supplementary material for: Impact of age and comorbidities on SARS-CoV-2 vaccine-induced T cell immunity
Source: Commun Med (Lond). 2023 Apr 24;3:58. doi: 10.1038/s43856-023-00277-x (PMC10124939; doi:10.1038/s43856-023-00277-x)
Supplement: Supplementary file 4 — Reporting Summary [file 43856_2023_277_MOESM4_ESM.pdf]

nature research

Corresponding author(s): Anna Karina Juhl (ANAJUH@rm.dk)
Lisa Loksø-Dietz (LISDIE@rm.dk)

Last updated by author(s): Jan 20, 2023

Reporting Summary

Nature Research wishes to improve the reproducibility of the work that we publish. This form provides structure for consistency and transparency in reporting. For further information on Nature Research policies, see our Editorial Policies and the Editorial Policy Checklist.

Statistics

For all statistical analyses, confirm that the following items are present in the figure legend, table legend, main text, or Methods section.

n/a	Confirmed	
☐	☒	The exact sample size (*n*) for each experimental group/condition, given as a discrete number and unit of measurement
☐	☒	A statement on whether measurements were taken from distinct samples or whether the same sample was measured repeatedly
☐	☒	The statistical test(s) used AND whether they are one- or two-sided<br>*Only common tests should be described solely by name; describe more complex techniques in the Methods section.*
☐	☒	A description of all covariates tested
☐	☒	A description of any assumptions or corrections, such as tests of normality and adjustment for multiple comparisons
☐	☒	A full description of the statistical parameters including central tendency (e.g. means) or other basic estimates (e.g. regression coefficient) AND variation (e.g. standard deviation) or associated estimates of uncertainty (e.g. confidence intervals)
☒	☐	For null hypothesis testing, the test statistic (e.g. *F*, *t*, *r*) with confidence intervals, effect sizes, degrees of freedom and *P* value noted<br>*Give P values as exact values whenever suitable.*
☒	☐	For Bayesian analysis, information on the choice of priors and Markov chain Monte Carlo settings
☒	☐	For hierarchical and complex designs, identification of the appropriate level for tests and full reporting of outcomes
☒	☐	Estimates of effect sizes (e.g. Cohen's *d*, Pearson's *r*), indicating how they were calculated

Our web collection on statistics for biologists contains articles on many of the points above.

Software and code

Policy information about availability of computer code

Data collection	Flow cytometry data collection: MACSQuant16 (Miltenyi Biotec)<br>Antibody data collection: MESO SECTOR S600 Reader
Data analysis	Flow analysis: FlowJo™ v10.8.1 Software (BD Biosciences)<br>Meso Scale analysis: MSD Discovery Workbench Software (Version 4.0)<br>Statistical analysis and visualization was done by Python version 3.9 using matplotlib and seaborn (logistic regression was done using the generalized linear model from R stats version 3.6.2). Tables were created created with tableone version 0.7.10 in Python.

For manuscripts utilizing custom algorithms or software that are central to the research but not yet described in published literature, software must be made available to editors and reviewers. We strongly encourage code deposition in a community repository (e.g. GitHub). See the Nature Research guidelines for submitting code & software for further information.

Data

Policy information about availability of data

All manuscripts must include a data availability statement. This statement should provide the following information, where applicable:
- Accession codes, unique identifiers, or web links for publicly available datasets
- A list of figures that have associated raw data
- A description of any restrictions on data availability

Data from the ENFORCE cohort may be made available to researchers upon approval of an application to retrieve data by the ENFORCE scientific steering committee. If approval is granted data will be provided as deidentified data. The ENFORCE protocol is available at www.enforce.dk.

Field-specific reporting

Please select the one below that is the best fit for your research. If you are not sure, read the appropriate sections before making your selection.

☒ Life sciences ☐ Behavioural & social sciences ☐ Ecological, evolutionary & environmental sciences

For a reference copy of the document with all sections, see nature.com/documents/nr-reporting-summary-flat.pdf

Life sciences study design

All studies must disclose on these points even when the disclosure is negative.

Sample size

The ENFORCE T cell immunity substudy aimed to enroll 10% of the total ENFORCE participants, this resulted in a cohort of 655 participants. In the clinical protocol it states the following regarding the rational of the sample size:
"A total of 2500 persons per vaccine will be included. Table 1 shows the minimum equivalence margin (δ) that can be attained with 90% certainty when comparing two vaccine groups for varying vaccine efficacy levels (% achieving MPNAT) and sample sizes. Computations are based on simulating 10,000 datasets to compare two vaccines.
For a study comparing two vaccines with a sample size of 2,500 per group and with 90% achieving the MPNAT (the chosen measure of vaccine efficacy), it will be possible with 90% certainty to achieve equivalence, with an equivalence margin of δ= +/-2.79%. Similarly, for a vaccine where only 70% achieve the MPNAT, the corresponding minimum equivalence margin that could be attained is δ= +/-4.26%. Thus, from table 1 with a sample size of 2,500 per group our simulations show that with an equivalence margin set at δ=+/-5% we will have sufficient power to ascertain equivalence for all titre levels with ≥90% certainty.

At smaller sample sizes the minimum δ that can be evaluated to assess equivalence with 90% certainty is larger. For example, when only 500 participants are included per group, the minimum equivalence margins are δ =+/-8.87% and δ=+/-13.54% for vaccines where 90% and 70%, respectively, achieve the MPNAT.
The sample size computations outlined in table 1 are based on comparing two groups. The study will include four groups leading to six unique comparisons, and the final analysis should therefore allow for multiple testing. Further, such comparisons are not independent of each other and we will investigate how to allow for this so as to best preserve the precision of the equivalence trial. This will be a natural extension of the current simulation based approach."

Data exclusions

In the present study, participants with a SARS-CoV-2 infection prior to baseline, defined as positive SARS-CoV-2 PCR test or Spike Ig positive at baseline visit (WANTAI assay), were excluded from the analysis. A total of 699 participants were enrolled in the ENFORCE T cell immunity substudy. Due to prior SARS-CoV-2 infection, 44 were excluded. Thus, the study cohort consisted of 655 participants. Of the 655 participants included in the study, blood samples for analysis of SARS-CoV-2 Spike antibody profiling were collected for 560 (99.2%), 598 (91.3%), and 523 (79.8%) participants at baseline (day 0), day 21, and day 90, respectively. Blood samples for analysis of SARS-CoV-2 Spike-specific T cells with the activation induced markers (AIM) assay were collected for 638 (97.4%), 573 (87.5%), and 502 (76.6%) participants at baseline (day 0), day 21, and day 90, respectively. Logistic challenges in the beginning of study enrolment, led to poor sample quality for a number of baseline samples for analysis with the AIM assay. As most participants enrolled early in the study received the ChAdOx1 vaccine, all baseline samples in this vaccine group were lost. Thus, 286 and 272 of the 638 baseline samples met the quality criteria set for the AIM assay for CD4+ or CD8+ Spike-specific T cells, respectively. Of the 573 samples analysed at day 21, 460 (CD4+ T cells) and 444 (CD8+ T cells) met the quality criteria. Lastly, of the 502 samples at the day 90 visit, 462 (CD4+ T cells) and 449 (CD8+ T cells) met quality criteria. Samples that did not meet quality criteria were excluded from all data analysis.

Replication

Data acquisition was performed once per participant per analysis per visit. For some participants it would be possible to replicate analysis depending on sample availability from the biobank.

Randomization

The vaccine distribution was decided by the Danish government, and thus out of our control.

Blinding

The investigators were blinded to participant group allocation and all other participant information during data acquisition and analysis. When collecting samples for analysis and reporting data, participant IDs which in it self did not contain any information on the participant.

Reporting for specific materials, systems and methods

We require information from authors about some types of materials, experimental systems and methods used in many studies. Here, indicate whether each material, system or method listed is relevant to your study. If you are not sure if a list item applies to your research, read the appropriate section before selecting a response.

Materials & experimental systems

n/a	Involved in the study	
☐	☒	Antibodies
☒	☐	Eukaryotic cell lines
☒	☐	Palaeontology and archaeology
☒	☐	Animals and other organisms
☐	☒	Human research participants
☐	☒	Clinical data
☒	☐	Dual use research of concern

Methods

n/a	Involved in the study	
☒	☐	ChIP-seq
☐	☒	Flow cytometry
☒	☐	MRI-based neuroimaging

Antibodies

Antibodies used

CD3-PerCP-Cy5.5 (cat#BL344808, BioLegend), CD4-BV650 (cat# BL300536, BioLegend), CD8-BV605 (cat# BL301040, BioLegend), CD69-APC (cat#BL310910, BioLegend), OX40-BV421 (cat# BL 350014, BioLegend), 41BB-PE (cat# BL309804, BioLegend).

Validation

CD3-PerCP-Cy5.5 (cat#BL344808, BioLegend): Isotype Mouse IgG1, κ. (McDonnell A, et al. 2017. BMC Cancer . 10.1186/s12885-017-3403-5.) more information: https://www.biolegend.com/ja-jp/products/percp-cyanine5-5-anti-human-cd3-antibody-6932.

CD4-BV650 (cat# BL300536, BioLegend), isotype Mouse IgG1, κ (Catakovic K, et al. 2018. Oncoimmunology. 7:e1371399). For more information: https://www.biolegend.com/ja-jp/products/brilliant-violet-650-anti-human-cd4-antibody-7650.

CD8-BV605 (cat# BL301040, BioLegend), Isotype Mouse IgG1, κ. (Johnson L, Olson B, and McNeel D. 2017. J Immunother Cancer. 10.1186/s40425-017-0260-3.) For more information go to: https://www.biolegend.com/ja-jp/products/brilliant-violet-605-anti-human-cd8a-antibody-7651

CD69-APC (cat#BL310910, BioLegend): Isotype Mouse IgG1, κ. (Jeng MY, et al. 2018. J Exp Med. 215:51.) for more information go to https://www.biolegend.com/ja-jp/products/apc-anti-human-cd69-antibody-1674

OX40-BV421 (cat# BL 350014, BioLegend): Isotype Mouse IgG1, κ (Robinson A, et al. 2014. Am J Physiol Gastrointest Liver Physiol. 307:1115.) For more information go to: https://www.biolegend.com/ja-jp/products/brilliant-violet-421-anti-human-cd134-ox40-antibody-7335

41BB-PE (cat# BL309804, BioLegend): Isotype Mouse IgG1, κ (Gordon-Alonso M,et al. 2017. Nat. Commun. 10.1038/s41467-017-00925-6.) For more information go to: https://www.biolegend.com/ja-jp/products/pe-anti-human-cd137-4-1bb-antibody-1510

Human research participants

Policy information about studies involving human research participants

Population characteristics

A total of 699 participants were enrolled in the ENFORCE T cell immunity substudy. Due to prior SARS-CoV-2 infection, 44 were excluded. Thus, the study cohort 79 consisted of 655 participants (56.3% females) with a median age of 63 years (IQR: 51–72). The majority received two doses of BTN162b2 (46.8%, n=314), or two doses of mRNA-1273 (37.4%, n=251), while 15.8% (n=106) received one dose of ChAdOx1 followed by a second dose of either of the two mRNA vaccines. Individuals who received BTN162b2 had a higher prevalence of comorbidities and a higher median age (71 years) than both mRNA-1273 and ChAdOx1 recipients (median ages of 62 and 50 years, respectively). Moreover, participants receiving ChAdOx1 were predominantly young female healthcare workers with very few comorbidities (See Table 1 in the paper).

Recruitment

The National Cohort Study of Effectiveness and Safety of SARS-CoV-2 vaccines (ENFORCE) is a Danish open-label Phase IV study, which is non-randomized with parallel groups. The study enrolled Danish citizens prior to vaccination against COVID-19. Participants were invited

Ethics oversight

The study protocol was approved by the Danish Medicines Agency (#2020-006003-42), and the National Committee on Health Research Ethics (#1-10-72-337-20).

Note that full information on the approval of the study protocol must also be provided in the manuscript.

Clinical data

Policy information about clinical studies

All manuscripts should comply with the ICMJE guidelines for publication of clinical research and a completed CONSORT checklist must be included with all submissions.

Clinical trial registration

clinicaltrials.gov, identifier: NCT04760132

Study protocol

The ENFORCE protocol is available at www.enforce.dk.

Data collection

The second study visit (study visit day 21) occurred 0-7 days prior to the second vaccine dose (median of 21, 34, and 84 days after the

Data collection	first vaccine dose for BTN162b2, mRNA-1273, and ChAdOx1, respectively). The third study visit (study visit day 90) occurred 90 days (+/-14 days) after the first vaccine dose (a median of 91 days for BTN162b2 and mRNA-1273, and 100 days for ChAdOx1). Blood samples for measuring SARS-CoV-2 Spike-specific T cells and IgG levels were obtained at each study visit.
Outcomes	The present study was a predefined ENFORCE T cell immunity substudy, which was part of the master protocol of the ENFORCE study. The primary objective of the substudy is determination of cellular immunity following COVID-19 vaccination among a subset of ENFORCE participants.

Flow Cytometry

Plots

Confirm that:

☒ The axis labels state the marker and fluorochrome used (e.g. CD4-FITC).

☒ The axis scales are clearly visible. Include numbers along axes only for bottom left plot of group (a 'group' is an analysis of identical markers).

☒ All plots are contour plots with outliers or pseudocolor plots.

☒ A numerical value for number of cells or percentage (with statistics) is provided.

Methodology

Sample preparation	Purified peripheral blood mononuclear cells (PBMCs) were stimulated with PepMix™ SARS-CoV-2 (product code PM-WCPV-S-1) at 2µg/ml or negative control (Dimethyl sulfoxide) for 20 hours. Following stimulation, the cells were washed and stained.
Instrument	MACSQuant16 (Miltenyi Biotec)
Software	FlowJo™ v10.8.1 Software (BD Biosciences)
Cell population abundance	We did not use flow cytometry to sort cells, thus this is not applicable.
Gating strategy	Live cells were gated by the dead cell stain as the negative population. Single cells were gated in a FSC-A/FSC-H plot. Lymphocytes were gated in a FSC-A/SSC-A plot. CD3+ cells (T lymphocytes) were gated by CD3 positivity. CD4+ and CD8+ T cells were gated as single positive for either CD4 or CD8, respectively. Lastly both CD4+ and CD8+ cells were gated for the three AIMs (Supplementary Figure 6). Boolean gating for the three AIMs was performed on both CD4+ and CD8+ T cells to identify double- and triple positive cells. Lastly, background was subtracted from Spike stimulated cells to get the final percentage of SARS CoV-2 Spike-specific T cells. Samples with negative values (i.e., where background signal was higher than signal in Spike stimulated cells) were turned to zero. Data was excluded if either the viability of the sample was below 70% at flow data acquisition or if the CD4+ and/or CD8+ T cell count was below 10,000.

☒ Tick this box to confirm that a figure exemplifying the gating strategy is provided in the Supplementary Information.
